# Supplementary material for: Curated and harmonised transcriptomics datasets of interstitial lung diseases
Source: Data Brief. 2025 Oct 14;63:112139. doi: 10.1016/j.dib.2025.112139 (PMC12581653; doi:10.1016/j.dib.2025.112139)
Supplement: Supplementary file 1 [file mmc1.zip › Supplementary_material/RNA-seq/fastQC reports/GSE124685/SRR8397173_fastqc.html]

SRR8397173.fastq.gz FastQC Report 

FastQC Report

Tue 30 Jan 2024  
SRR8397173.fastq.gz

## Summary

- Basic Statistics
- Per base sequence quality
- Per sequence quality scores
- Per base sequence content
- Per sequence GC content
- Per base N content
- Sequence Length Distribution
- Sequence Duplication Levels
- Overrepresented sequences
- Adapter Content

## Basic Statistics

| Measure | Value |
| --- | --- |
| Filename | SRR8397173.fastq.gz |
| File type | Conventional base calls |
| Encoding | Sanger / Illumina 1.9 |
| Total Sequences | 26943749 |
| Sequences flagged as poor quality | 0 |
| Sequence length | 8-368 |
| %GC | 53 |

## Per base sequence quality

## Per sequence quality scores

## Per base sequence content

## Per sequence GC content

## Per base N content

## Sequence Length Distribution

## Sequence Duplication Levels

## Overrepresented sequences

| Sequence | Count | Percentage | Possible Source |
| --- | --- | --- | --- |
| GGGCCACGAGCTGAGTGCGTCCTGTCACTCCACTCCCATGTCCCTTGGGA | 356952 | 1.3248045028923037 | No Hit |
| AAGCTCAGGGAGAGCCCTGTTAGGGCCGCCTCTGGCCCTAGTCTCAGACC | 345326 | 1.2816553479621562 | No Hit |
| ACACTGAGGACTCTGTTCCTCCCCTTTCCGCCTAGGGGAAAGTCCCCGGA | 280264 | 1.0401818989629097 | No Hit |
| GGGAATGTCTACGTGCGTATGCACGTGGCACTCTCTGCCCGAGGTCCGGG | 248959 | 0.9239953949986692 | No Hit |
| AGCGGTTCCGCTGCCCTGAGGCACTCTTCCAGCCTTCCTTCCTGGGCATG | 141710 | 0.5259475954886604 | No Hit |
| CCACCTTCTCCATCGGCTCCACTGGCCTCGTGGTGTATGACTACCAGCAG | 141323 | 0.5245112697568552 | No Hit |
| TCACACTTCATGATGGAGTTGAAGGTAGTTTCGTGGATGCCACAGGACTC | 134536 | 0.49932175362827197 | No Hit |
| TCTCTGGGCTTCTATTTCGACCGCGATGATGTGGCTCTGGAAGGCGTGAG | 134447 | 0.4989914358243168 | No Hit |
| AAAAGTGATGGACTGCCCATTGCTGGAGAAGACCTTCTCTCCTACTGTCA | 119600 | 0.44388774553979105 | No Hit |
| ATGATGTAGCAGCAGGTGCCAGGGGCTGGCTTGTAGGCGATCAGCAGCTG | 115803 | 0.4297954230497026 | No Hit |
| TCTTTCTGGCCTGGAGGCTATCCAGCGTACTCCAAAGATTCAGGTTTACT | 106817 | 0.39644445915822624 | No Hit |
| GGGTACTTCAGGGTCAGGATGCCACGCTTGCTCTGGGCCTCGTCGCCCAC | 101255 | 0.37580145212902627 | No Hit |
| ATCAAATCCTGCAGACAAGGGGAGCCCTCAGTCTGCAGGGCTCCATAATG | 94276 | 0.34989934028853964 | No Hit |
| TGGTTCCTGGCAAGCCCATGTGTGTTGAGAGCTTCTCAGACTATCCACCT | 86888 | 0.3224792511242589 | No Hit |
| TGAGGCGGAGCACCAGAGAGCCTACCTGGAAGACACATGCGTGGAGTGGC | 72735 | 0.2699512974233838 | No Hit |
| ACACCCACCGCAACTGTCTGTCTCATATCACGAACAGCAAAGCGACCCAA | 68692 | 0.2549459616774191 | No Hit |
| ACATAGCAATTCAGGAAATTTGACTTTCCATTCTCTGCTGGATGACGTGA | 64656 | 0.23996660598345093 | No Hit |
| AGCGGTTCCGGTGTCCGGAGGCGCTGTTCCAGCCTTCCTTCCTGGGTATG | 61952 | 0.2299308830408122 | No Hit |
| TCACACTTCATGATGGAGTTGAAGGTGGTCTCGTGGATGCCGCAAGATTC | 54482 | 0.2022064561245727 | No Hit |
| TCTGTCTTCTTCAGTTTCGACTTATCGAATTTCTCGATCTCAGCCATATC | 51667 | 0.19175876378598986 | No Hit |
| TCGGGCGCCCCAGACACCAGGCGTCATGGTGGGCATGGGCCAGAAGGACT | 46637 | 0.17309024070852205 | No Hit |
| TGTTCCCTCTCCTCATGAGATTGGTGAAGAAAGTATTTGGCAAAGTTCTT | 45891 | 0.1703215094528976 | No Hit |
| GGCTGCCTGGAGCCCCTGGTGTCCCTGGAGAGCGTGGAGAGAAGGGGGAG | 43199 | 0.16033032374225278 | No Hit |
| TGTCCATGTCTTACTACTTTGACCGCGATGATGTGGCTTTGAAGAACTTT | 39982 | 0.14839063413187228 | No Hit |
| ACGCTCGTAGCCCTCGCGCTTCTCCTCGGCCAATTCGCGGAAGAAGTGGC | 39447 | 0.1464050158721416 | No Hit |
| GGGGCTCCAGGTGAAGCAGCGTCTCCTTCCCCTTCTCCAGGTATTTGTGG | 39271 | 0.1457518031362302 | No Hit |
| GCTGCTACAGGAGAATAGCAGACAGGTATAGTTAAGAAACAACTTTATTT | 37170 | 0.1379540761012879 | No Hit |
| ACGCTCGTAGCCCTCGCGCTTCTCCTCGGCCAGTTCGCGGAAGAAGTGGC | 37077 | 0.1376089125533347 | No Hit |
| GGGAATGTCTACGTGCGTATGCACGTGGCACTCTCTGCCCGGAGGTCCGG | 36982 | 0.13725632613338254 | No Hit |
| CGTGCTGGCTACTACCGACCGGAGGCGCCGTGACCTTGGTGGCTCAGCCC | 36783 | 0.13651775036948274 | No Hit |
| GAGCTCCTCATCTAGATGAGCTGGAAGCCCTGGAGGGCCTCTCTCGCCAG | 36559 | 0.1356863887055955 | No Hit |
| CTAAGTGCTGTGTTGTCGTTCCCCCTGCTTAAAATAAAGTTGTTTCTTAA | 36121 | 0.134060779737816 | No Hit |
| TACTCGTGCGCCTCGCTTCGCTTTTCCTCCGCAACCATGTCTGACAAACC | 36025 | 0.13370448188186432 | No Hit |
| ACACTGAGGACTCTGTTCCTCCCCTTTCCGCCTAGGGAAAGTCCCCGGAC | 33145 | 0.12301554620331417 | No Hit |
| CCAGTGTAGTCAATAGCCAGGAGGTGTCCAAGGGCTACAGAGAGGCCGAT | 32978 | 0.12239573639139824 | No Hit |
| TCGGCGCCCCAGACACCAGGCGTCATGGTGGGCATGGGCCAGAAGGACTC | 32763 | 0.12159777765150648 | No Hit |
| TCAGCACGGGCTCGTACTCTGCCACAAACTGATCACACTGCTTCTGGTAA | 32641 | 0.1211449824595679 | No Hit |
| CCGGACTCCACCTGGCTGTTGTCAATCTTCACCTCATAGGTGTTGTCTGG | 30295 | 0.11243795360474891 | No Hit |
| CGGTTGTACTTGCGGATGTAGTGCAGATAGTCTCGGCGGATGACAATGGT | 29787 | 0.11055254411700464 | No Hit |
| GACATCCGTTGCAAGGATGATGAGTTTACACACCTGTACACACTGATTGT | 28712 | 0.10656275041754583 | No Hit |
| AGGATGCCCAGAATCAGGGCCCAGATGTTCAGGCACTTGGCGGTGGAGGC | 28060 | 0.10414289414587406 | No Hit |
| GGGCTCCAGGTGAAGCAGCGTCTCCTTCCCCTTCTCCAGGTATTTGTGGA | 27756 | 0.10301461760202711 | No Hit |
| ACCTACGGCGAGGGCGAGAGCGGCCCCATGGGCAACATCATGATCGATCC | 27616 | 0.10249501656209759 | No Hit |

## Adapter Content

Produced by FastQC (version 0.11.7)
